# EXPOSOMES and GENES: The duo influencing CANCER initiation and progression

**DOI:** 10.18632/oncotarget.28696

**Published:** 2025-03-10

**Authors:** Uzma Saqib, Katherine E. Ricks, Alexander G. Obukhov, Krishnan Hajela

**Keywords:** cancer, exposomes, genes, food

Genes are the essential blueprints storing the instructions for protein biosynthesis in our body. Errors in the blueprints may have deleterious consequences. For example, congenital gene mutations may lead to diseases, such as cystic fibrosis (*CFTR* gene mutations), Duchenne muscular dystrophy (*DMD* gene mutations), or early onset cancers (*BRCA1* and *BRCA2* mutations). Furthermore, sporadic mutations in *NF1, PTPN11, SOS1, SOS2, RAF1, RIT1, KRAS, NRAS, SHOC2, RRAS, BRAF, HRAS* among other genes [1], can initially appear in one single cell. This cell can then divide to form a tumour or spread throughout the body, potentially giving rise to a metastasizing cancer. These spontaneous mutations can result from exposure to harsh environmental factors, such as X-ray radiation, solar radiation, cigarette smoke, or mutagenic chemicals. Additionally, the environment can affect differential expression of genes in our cells. This is critical for our survival because we need to adapt to environmental changes. However, some changes in gene expression may also cause health problems. The complex combination of all environmental factors acting on our body throughout the entire lifespan is referred to as the *exposome*. This term was coined by Dr. Wild [2] in 2005 and encompasses all environmental exposures, including diet, air pollution, solar and cosmic radiation, tobacco smoke exposure, stress, intestinal microbiome changes, bacterial, parasitic or viral infections, and other elements that people are subjected to throughout their lifetime. The unilateral effect of exposomes on human genes and consequently human health has recently gained attention because of its role in cancer initiation and progression. Environmental exposures may cause genetic damage, lead to mutations in key genes, and/or block the DNA repair mechanisms increasing the risk of cancer. Researchers across the globe are characterizing how exposomes influence genes and cancer development.

It has been estimated that 30–40% of cancers can be prevented with an active lifestyle and good dietary practices [3]. For example, nasopharyngeal cancer is common in the world populations that relies on the consumption of processed foods [4]. These foods contain preservatives, such as nitrosamine derivatives, which are known to impact gene expression. The International Agency for Research on Cancer (IARC) classified processed meat as carcinogenic because it contains mutagenic N-nitroso compounds (https://publications.iarc.fr/Book-And-Report-Series/Iarc-Monographs-On-The-Identification-Of-Carcinogenic-Hazards-To-Humans/Red-Meat-And-Processed-Meat-2018). Conversely, the excessive consumption of alcoholic beverages has been shown to promote hepatocarcinogenesis, likely due to cellular accumulation of acetaldehyde, a toxic alcohol metabolite increasing the point mutation frequency. Likewise, a sedentary lifestyle has been associated with obesity and higher plasma levels of metabolites capable of stimulating the epigenetic pathways leading to cancer progression.

According to the Global Air Quality Guidelines of World Health Organization (WHO), nearly all of the global population (>99%) breathes polluted air that exceeds guideline limits (ISBN: 9789240034228). Air pollution encompasses such chemicals as particulate matter (PM), carbon monoxide, ozone, nitrogen dioxide, and others. Many studies show a direct link between PM and epidermal growth factor receptor (EGFR) mutations, leading to lung cancer development. UV light exposure-induced DNA modification is a leading cause of skin cancer worldwide. Water polluted by organic mutagenic compounds (polycyclic aromatic hydrocarbons) may also cause various human diseases including cancer. Arsenic present in food and water is one of the major contributors to cancer initiation via DNA methylation. Thus, each of these exposures modifies DNA, potentially contributing to cancer development.

Stress hormones have also been directly linked to cancer development. For example, corticosteroids inhibit the tumour suppressor protein p53 via genetic changes [5]. Similarly, catecholamines activate epigenetic activators that are associated with promoting the migration of colon carcinoma cells and stimulate the G-protein related signalling pathways triggering DNA damage. Thus, chronic stress may facilitate cancer initiation and progression.

Exposure to external environmental factors like bacteria, virus, and fungi has been suggested to cause cellular damage leading to human cancers. For example, H*. pylori* can dysregulate host intracellular signalling pathways and lead to neoplastic transformation and gastric cancer development via injecting its *cagA* gene products into the host gastric epithelial cells, leading, in part, to host gastric epithelial cell genome instability [6]. Conversely, *S. typhi* and *C. pneumoniae* infections cause inflammation and reactive oxygen species (ROS)-dependent DNA damage leading to gallbladder cancer [7] and lung cancer [8], respectively. Human Papilloma Virus (HPV) infection has been strongly associated with cervical cancers. The E6 and E7 proteins of mucosal high-risk HPV have a transforming ability to cause genetic abnormalities such as monosomies, trisomies, chromatid gaps and breaks [9]. Genome instability linked to ROS overproduction was also noted in Hodgkin’s lymphoma, Burkitt’s lymphoma, Nasopharyngeal carcinoma after Epstein–Barr virus (EBV) infection [10]. On the other hand, fungal infections with *Trichosporon, Fusarium, Rhizopus, H. capsulatum, H. immitis,* and *C. neoformans* have been associated with epigenetic changes leading to lung cancer [11].

Exposomes differ depending on the location, occupation, and lifestyles of individuals. It becomes increasingly difficult for researchers and policymakers to draw robust conclusions about the effects of exposomes on specific populations because it is a challenging job to characterise exposomes. Technologies like Chemical Isotope Labeling Exposome (CIL-EXPOSOME) [12] and high-throughput methods (e.g., ‘omics’ technologies) allow measuring multiple exposures at once [13]. The National Health and Nutrition Examination Survey (NHANES), a biannual health survey conducted by US Centers for Disease Control and Prevention, measures factors including environmental exposures like chemicals, nutrients, and infectious agents in human tissue samples and fluids (https://www.cdc.gov/nchs/nhanes/index.htm). Quantitative measurements of nutrients and pollutants in human tissues are done using comprehensive analytical techniques such as liquid chromatography and gas chromatography, while infectious agents are measured via immunological assays. Self-reported information is also collected on nutrient consumption, physical activity, and prescribed pharmaceutical drugs. Mass Spectrometry is used to determine small molecule chemical elements of exposomes, whereas ‘omics’ technologies are useful for quantification of environmental chemicals in human fluids and tissue samples.

**Figure 1:**
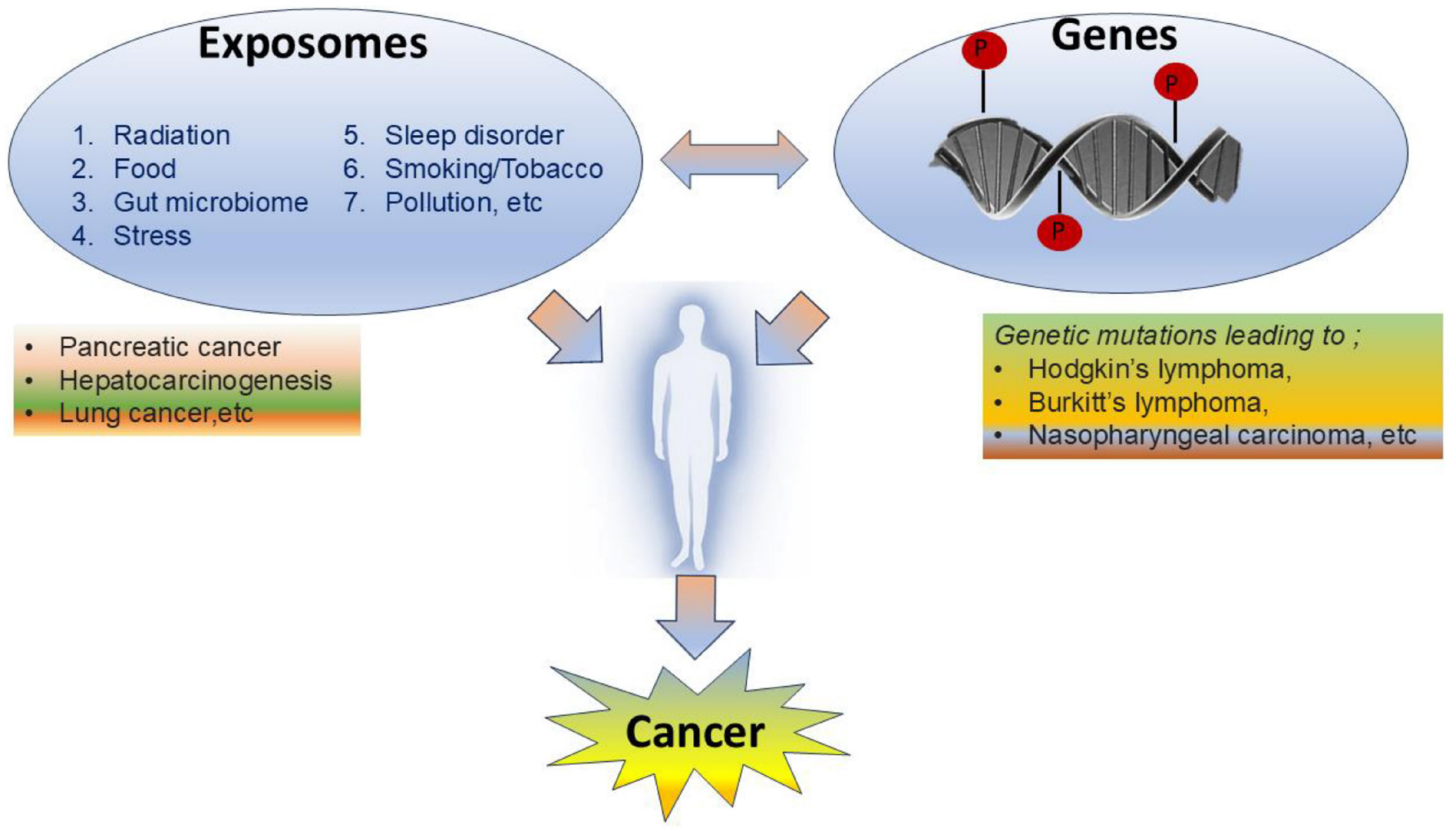
The correlation between exposomes, genes and cancer.

Understanding the exposome-gene-cancer research axis will have a significant impact on public health and the development of more effective strategies for prevention and treatment of diseases (Figure 1). Further research is needed to better understand how environmental exposures impact gene expression and contribute to cancer development. Identifying novel biomarkers and therapeutic targets are critical for cancer diagnosis and prevention. Exploration of exposomes will allow policymakers to proactively direct their legislative efforts for eliminating the dangerous elements of the exposomes. The information on the impact of exposomes on human health should be popularized to help each individual to make better choices for their health. As scientists continue to explore varied exposure assessment methods, identify novel biomarkers, and develop sophisticated analytical approaches, the exposome research is positioned to revolutionize the fields of public health specifically with respect to cancer prevention.
